# PAIN ASSESSMENT AND MANAGEMENT FOR OLDER ADULTS WITH DEMENTIA IN LONG-TERM CARE: A SCOPING REVIEW

**DOI:** 10.1093/geroni/igad104.2550

**Published:** 2023-12-21

**Authors:** Minhee Yang, Jeongwon Shin

**Affiliations:** Yonsei University, Seoul, Republic of Korea; Yonsei University, Seoul, Republic of Korea

## Abstract

Self-reporting is essential for assessing pain. However, owing to their cognitive impairment, older adults with dementia may not always express pain. As such, older adults with dementia are at risk of living with undertreated pain. This scoping review aimed to explore and identify current publication trends and strategies regarding pain assessment and management for older adults with dementia in long-term care. Using the scoping review method of Arksey and O’Malley, two reviewers conducted structured searches for articles published from 2012 to 2022 on four databases (CINAHL, Cochrane, PubMed, RISS) and then independently reviewed the full text for eligibility criteria. The review included 40 articles for analysis and identified 20 observational pain tools used in the studies. A few studies assessed pain in older adults with dementia using facial expression recognition technology and electroencephalography. A common pain management strategy identified for older adults with dementia was the application of protocols or clinical guidelines that included a pain assessment and treatment phase. In addition to pharmacological and non-pharmacological treatment, a therapeutic robot (PARO) and play activities were investigated to determine whether the pain was alleviated in older adults with dementia. Although studies have been conducted to assess and manage pain in older adults with dementia in long-term care, the review highlighted the need for education and training on pain tools and treatment approaches, as well as for the development of pain management strategies and non-pharmacological interventions.

